# Supplementary material for: Resolution of superluminal signalling in non-perturbative cavity quantum electrodynamics
Source: Nat Commun. 2018 May 15;9:1924. doi: 10.1038/s41467-018-04339-w (PMC5954151; doi:10.1038/s41467-018-04339-w)
Supplement: Supplementary file 1 — Supplementary Information [file 41467_2018_4339_MOESM1_ESM.pdf]

Supplementary Information:
Resolution of superluminal signalling in non-perturbative cavity quantum electrodynamics

Sánchez Muñoz *et al.*

SUPPLEMENTARY NOTE 1: DECAY OF THE OVERLAP $O_N(t)$

As discussed in the main body of the paper, the dynamical features that we have reported are a function of the value of the cutoff N chosen. In particular, the larger the number of modes we consider (larger N), the lower g/ω_c needs to be for the single-mode physics to break down and the evolution to consist of a succession of sharp revival peaks. The key of this observation lies in $O_N(t)$, the overlap between the two multi-mode cavity states $|\mp\xi_N(t)\rangle$ that evolve in association with the two qubit states, $|\pm\rangle$. As this overlap tends to zero, so does the coupling between the qubit states $|+\rangle$ and $|-\rangle$ induced by the Hamiltonian term $\omega_x\sigma_z/2$. As can be seen in Supplementary Figure 1, the evolution of $O_N(t)$, given by the exponential of Eq. (10) in the main body of the paper, consists of a rapid decay, on a timescale $\tau \approx 2\pi/(\omega_c N)$ mostly independent from g, to a stationary value approximately given by

$$\bar{O}_N \approx 1/[2e^{\gamma}(N+1)]^{4g^2/\omega_c^2}\,, \tag{1}$$

(where γ is the Euler-Mascheroni constant) that tends to zero with N. Supplementary Equation (1) is obtained from the evaluation of Eq. (10) at the middle point between revivals, $t/(2\pi/\omega_c) = 1/2$, and it shows the impact of N in making the overlap vanish to be logarithmic when compared to the effect of the coupling strength g. To give a more qualitative idea of the dependence of $\bar{O}_N$ on g, we plot it in Supplementary Figure 2(a) for values of N going from 10 to 100. For values of $g/\omega_c \ll 1$, we see that $\bar{O}_N$ tends to zero sublinearly with N. In the realistic range of tens to hundreds of cavity modes that we consider here, the variation of $\bar{O}_N$ with N can thus be neglected, as one can appreciate from the accumulation of curves in Supplementary Figure 2(a).

SUPPLEMENTARY NOTE 2: BREAKDOWN OF THE SINGLE-MODE PHYSICS

We have shown that the multi-mode dynamics characterized by the collapse and revival peaks on $O_N(t)$—and consequently on the population of the TLS—is directly linked to the propagation of photonic wavefronts inside the cavity. This effect appears immediately when one disregards the Hamiltonian term $H_{\rm II} = \omega_x\sigma_z/2$. Therefore, we will talk of a breakdown of the single-mode physics whenever this term does not play a role in the dynamics if a few modes are involved, but it does in the single-mode case $N = 1$. Even with $N = 1$, such a regime of collapse and revivals can be reached if g is well within the deep coupling regime $g/\omega_c > 1$, as was reported in [1] and is clear from our calculations and analytical expressions for $O_N(t)$. The novelty of our analysis is to reveal that, even with just a few cavity modes involved, this regime emerges at values of the coupling already in the ultrastrong coupling regime, $0.1 < g/\omega_c < 1$, which can be understood as being enforced by relativistic causality.

For $\omega_x = \omega_c$, a number of cavity modes $N > 10$ already ensures that the fast decay of $O_N(t)$—occurring on the timescale $\tau \approx 2\pi/(\omega_c N)$— will not be affected by such a term, i.e., the condition $N\omega_c \gg \omega_x$ is fulfilled. The breakdown of the single-mode physics will then occur when, considering the overlap between cavity states has already decayed to a stationary value $\bar{O}_N$, the coupling between the qubit states $|\pm\rangle$ through the $\omega_x\sigma_z/2$ term is sufficiently reduced by such overlap, i.e., when $\bar{O}_N\omega_x \ll g$. The ratio $g/(\bar{O}_N\omega_x)$ versus g is shown in Supplementary Figure 2(b), for the same range of N as in Supplementary Figure 2(a). We can mark the onset of multi-mode effects when this ratio becomes larger than one, and therefore define a critical coupling rate g_c as the one which fulfills $g_c/[\bar{O}_N(g_c)\omega_x] = 1$. Beyond this coupling rate, that will depend on N, we can

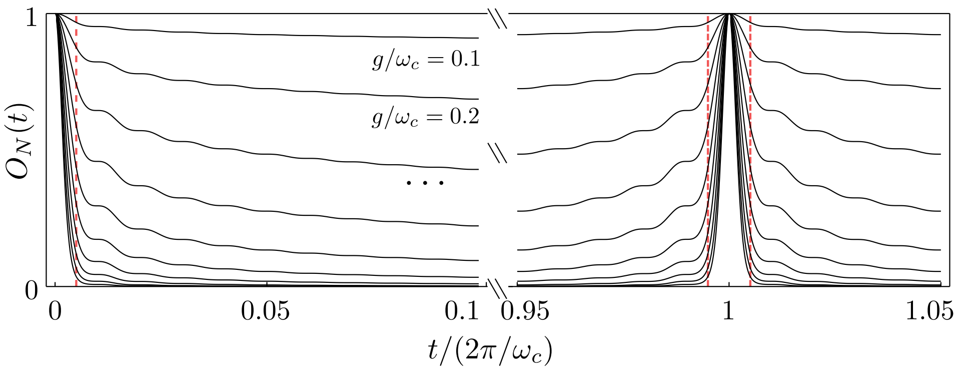

Supplementary Figure 1. Overlap $O_N(t)$ between the two cavity states $|\mp\xi_N(t)\rangle$ associated to the qubit states $|\pm\rangle$ versus time, for $N = 100$ and values of g/ω_c increasing from 0.1 to 1. Red gridlines are displayed at times $t/(2\pi/\omega_c) = (\sigma/2, 1\pm\sigma/2)$, with $\sigma = 1/N$. The timescale of the decay of overlap is not significantly dependent on g.

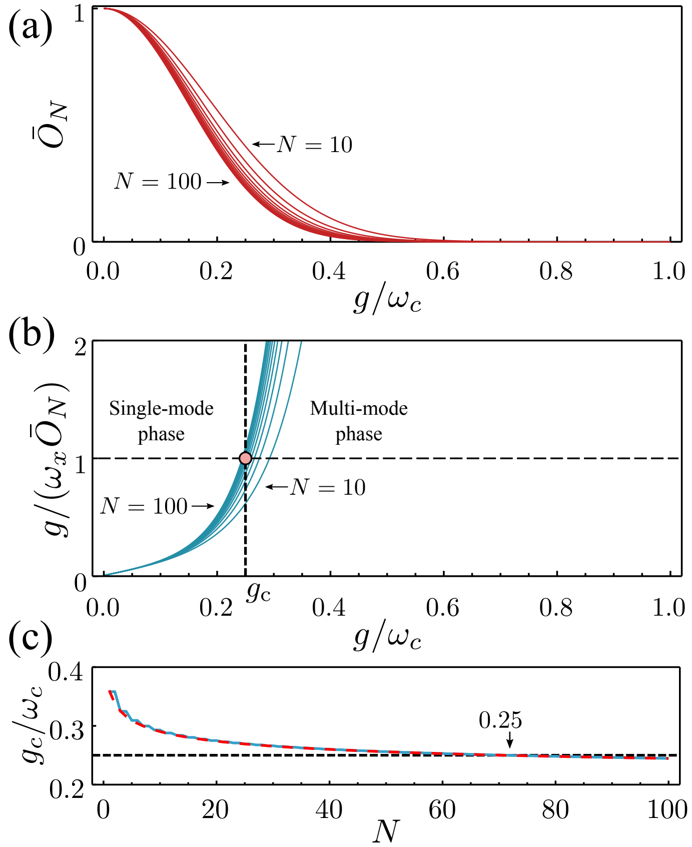

Supplementary Figure 2. (a) Steady overlap $\bar{O}_N$ versus the normalized coupling rate g/ω_c for values of N going from 10 to 100. (b) Ratio between coupling rate g and $\omega_x \bar{O}_N$. A ratio equal to one marks the onset of the multi-mode physics, provided that N is large enough so that, also, $N\omega_c \gg \omega_x$. (c) Normalized critical coupling rate versus N. The convergence to zero is slow, and in the range considered in this text, $g_c/\omega_c \approx= 0.25$. Solid-blue: numerical calculation. Dashed-red: analytical estimation from Supplementary Equation (2)

expect the single-mode physics to break down. By using Supplementary Equation (1), we can obtain the following approximate expression for g_c/ω_c:

$$\frac{g_c}{\omega_c} \approx \sqrt{\frac{W\{8\omega_x^2 \log[2e^\gamma(N+1)]/\omega_c^2\}}{8\log[2e^\gamma(N+1)]}}\,. \tag{2}$$

where $W(x)$ is the Lambert-W function. The dependence of g_c/ω_c with N is shown in Supplementary Figure 2(c). As N increases, g_c tends to zero very slowly, and for the range of N that we consider in this work, we find $g_c/\omega_c \approx 0.25$. This manifests clearly on Supplementary Figure 3, where we can observe how the single-mode model fails to describe the dynamics that emerge when one adds just a few number of modes, and this occurs approximately around the critical value $g/\omega_c \approx 0.25$ that we have obtained here.

SUPPLEMENTARY NOTE 3: LIGHT PROPAGATION IN MORE GENERAL MODELS

The results in the main body of the paper are obtained using a TLS approximation, which assumes that the emitter is well characterized by only two energy levels. The physics that we have described is, however, linked to the propagation of light. While the dipolar approximation can be used such a phenomenology is thus expected to be largely independent on the specific level-scheme of the emitter. In this section we demonstrate this explicitly considering, instead of a TLS emitter, a system in which the emitter consists of a nonlinear cavity with bosonic annihilation operator b and Kerr nonlinearity χ. The Hamiltonian reads:

$$H = \omega_x b^\dagger b + \chi b^\dagger b^\dagger b b + \sum_{n=0}^{N} \left\{\omega_c(n+1)a_n^\dagger a_n + g\sqrt{n+1}(a_n^\dagger + a_n)(b^\dagger + b)\right\}. \tag{3}$$

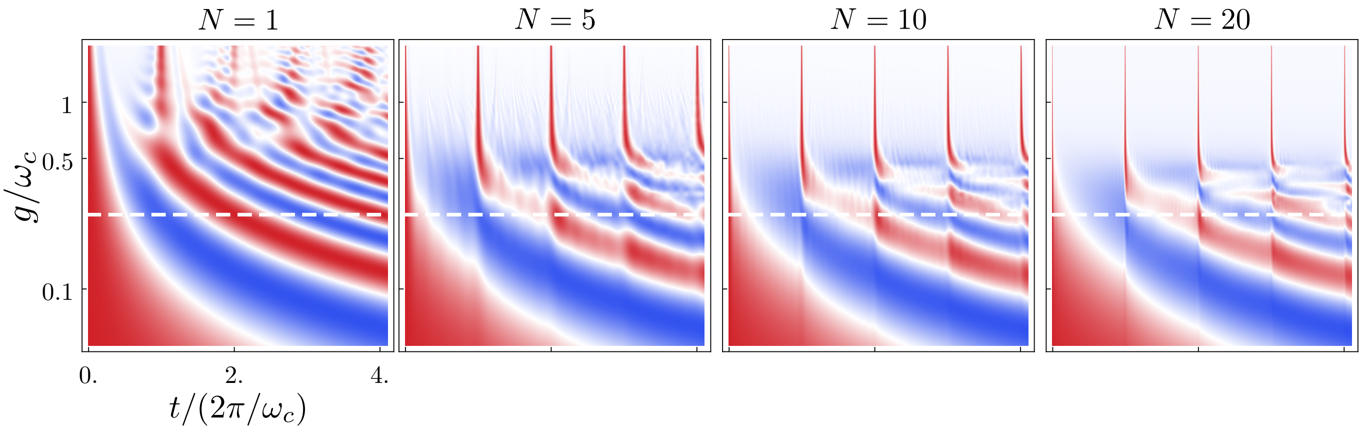

Supplementary Figure 3. Contour plot of the TLS population versus time and coupling rate for different values of N. The dashed line marks the critical coupling rate $g/\omega_c \approx 0.25$.

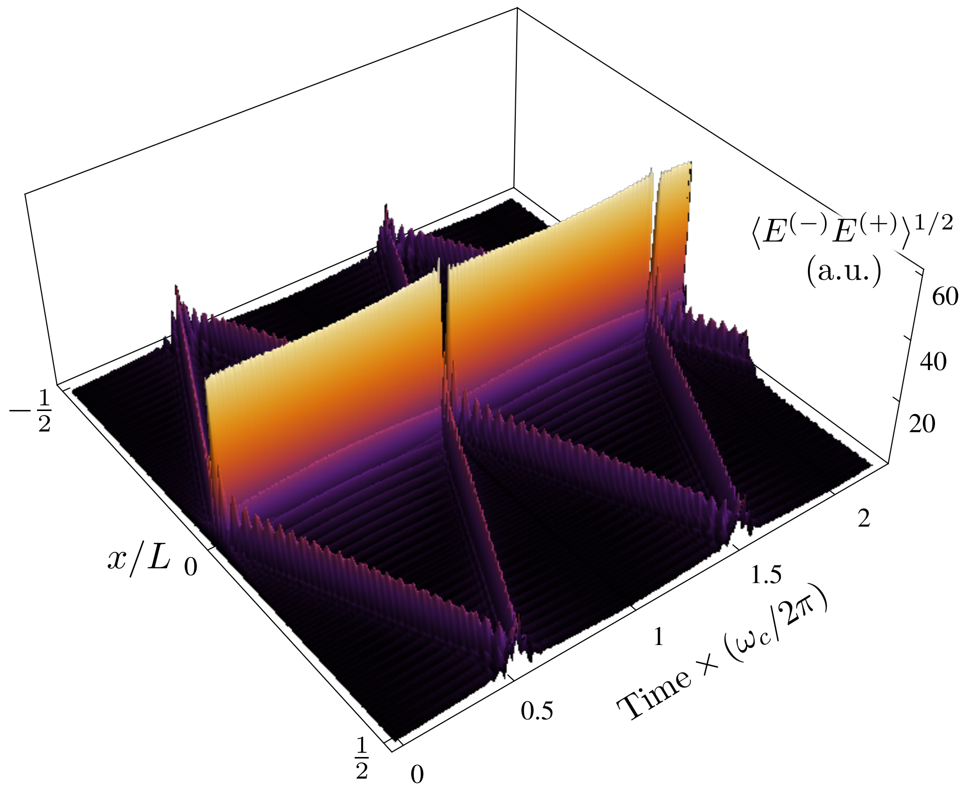

Supplementary Figure 4. Spatial profile of the electric field inside a cavity versus time, for an emitter consisting of a Kerr resonator. Parameters: $g/\omega_c = 0.6$, $\chi = 10\omega_c$.

The dynamics of this system, which can be computed by the method described in the main body of the paper, yields the same type of physics that we have presented so far. This is shown in Supplementary Figure 4, in which we chose a Kerr nonlinear coefficient small enough to have multiple electronic transitions resonantly coupled to the cavity photonic field. We see that the electric field inside the cavity features a localized photonic state bound to the emitter, and free propagating wavefronts. This calculation shows that our results, linked to light propagation in multi-mode systems, are robust and can manifest in a variety of systems.

SUPPLEMENTARY REFERENCES

[1] J. Casanova, G. Romero, I. Lizuain, J. J. García-Ripoll, and E. Solano, *Deep Strong Coupling Regime of the Jaynes-Cummings Model,* Phys. Rev. Lett. **105**, 263603 (2010).
